# Production of the First Effective Hyperimmune Equine Serum Antivenom against Africanized Bees 

**DOI:** 10.1371/journal.pone.0079971

**Published:** 2013-11-13

**Authors:** Keity Souza Santos, Marco Antonio Stephano, José Roberto Marcelino, Virginia Maria Resende Ferreira, Thalita Rocha, Celso Caricati, Hisako Gondo Higashi, Ana Maria Moro, Jorge Elias Kalil, Osmar Malaspina, Fabio Fernandes Morato Castro, Mário Sérgio Palma

**Affiliations:** 1 Division of Clinical Immunology and Allergy, University of São Paulo School of Medicine, FMUSP, São Paulo, Brazil; 2 Institute for Investigation in Immunology–INCT, São Paulo, SP, Brazil; 3 Faculdade de Ciências Farmacêuticas, São Paulo University, São Paulo, SP, Brazil; 4 Division of Hyperimmune Plasmas, Butantan Institute, São Paulo, SP, Brazil; 5 Medical School, São Francisco University, Bragança Paulista, SP, Brazil; 6 Special Pilot Laboratory of Research and Development of Veterinary Immunobiologicals, Butantan Institute, São Paulo, SP, Brazil; 7 Institute of Technology of Paraná, Curitiba, Paraná, Brazil; 8 Laboratory of Biopharmaceuticals in Animal Cells, Butantan Institute, São Paulo, SP, Brazil; 9 Heart Institute (InCor), LIM-19, University of São Paulo School of Medicine, São Paulo, SP, Brazil; 10 Department of Biology/Institute of Biosciences, Center for the Study of Social Insects, University of São Paulo State (UNESP), Rio Claro, SP, Brazil; Karolinska Institutet, Sweden

## Abstract

Victims of massive bee attacks become extremely ill, presenting symptoms ranging from dizziness and headache to acute renal failure and multiple organ failure that can lead to death. Previous attempts to develop specific antivenom to treat these victims have been unsuccessful. We herein report a F(ab)´_2_-based antivenom raised in horse as a potential new treatment for victims of multiple bee stings. The final product contains high specific IgG titers and is effective in neutralizing toxic effects, such as hemolysis, cytotoxicity and myotoxicity. The assessment of neutralization was revised and hemolysis, the primary toxic effect of these stings, was fully neutralized *in vivo* for the first time.

## Introduction

Africanized honeybees (AHB) derive from a founder population of the tropical-evolved African subspecies of *Apis mellifera scutellata* brought from South Africa into Brazil in 1956 to interbreed with previously imported European honeybees [1]. Some years later, a number of queens escaped by accident from the original apiary and survived in the wild in Brazil. These Africanized colonies quickly crossbred out of control with the common European sub-species of *A. mellifera* [1]. The highly defensive behavior of the Africanized bees and their astounding rate of spread contributed to an unusual capacity of moving great distances without intermediary colonization [2], favoring the dispersal of these wild colonies throughout the Americas until they reached the United States in 1990 [3]. The high aggressiveness of these colonies and their close contact with human populations resulted in a very high number of stinging accidents with serious medical consequences [4-7]. 

The frequency of mass bee attacks has dramatically increased in the Americas following the introduction and spread of the aggressive AHB, also known as the “killer bee”. According to the Brazilian Ministry of Health, the number of accidents involving these insects reached over 47,000 between the years 2000 and 2010 in Brazil, and these were associated with 153 deaths (Sinan/SVS/Brazilian Ministry of Health). In spite of this, no specific and safe therapy is currently available for the effective treatment of the victims of mass honeybee attacks. Currently, the administration of antihistamines, corticosteroids, bronchodilators, vasodilators, bicarbonate, mannitol, adrenaline and mechanical ventilation, in addition to hemodialysis sessions, are among the most prevalent non-specific therapies used to treat victims of multiple bee attacks; however, many of these therapies lack efficacy [8].

Honeybee venom did not evolve to be lethal, but, rather, to cause physical discomfort in the victim. Thus, depending on the number of stings received by the victim, the primary symptoms of envenomation are rhabdomyolysis, intravascular hemolysis, respiratory distress, hepatic dysfunction, myocardium damage, shock and renal failure [8,9]. Hypotension, tachycardia, respiratory distress, acute renal failure, disseminated intravascular coagulation and multiple organ dysfunction may also develop as delayed reactions [10]. Thus, honeybee envenomation is not usually lethal, but highly morbid. Victims of massive honeybee attacks may remain under intensive medical care for several days, during which the patients alternate between different periods of venom absorption and elimination [8]. If one considers that the elimination of venom occurs mainly in the liver and kidneys, these organs constitute the main target of tissue lesions due to prolonged contact with venom toxins. It is relatively common to observe chronic diseases of the kidneys and/or liver after a toxic shock caused by honeybee venom [9].

Therefore, there is an urgent need to develop honeybee antivenom, to provide an effective treatment for the victims of multiple stings. Only two reports in the literature describe the development of specific honeybee antivenom. Ovine bee F_ab_-based antivenom with positive neutralization results against PLA_2_ and European bee venom lethality (*Apis mellifera mellifera*) has been reported [11], but there have been no further reports of additional assays or any follow-up on this antivenom in the literature. Elsewhere, antibodies against PLA_2_ and melittin have been raised in rabbits, but they were ineffective in neutralizing whole venom lethality either alone or in combination [12]. These findings suggest that the reason for the ineffectiveness of these antibodies may be the type of animal system used to produce them. Therefore, other animal models should be tested to produce specific antibodies that can neutralize melittin activity. 

Taking advantage of the Brazilian experience in producing equine hyper-immune sera against other animal venoms, as well as our expertise in honeybee biology and the production of large quantities of honeybee venom, we created a consortium of academic and technological institutions to develop efficient equine anti-honeybee-venom hyperimmune serum. In the present study, we report the production, evaluation and preclinical assessment (*in vitro* and *in vivo*) of a specific horse antibody fragment (Fab´)_2_-based antivenom that will provide appropriate treatment for mass attacks by Africanized *A. mellifera*. This antivenom neutralized the main toxic effects of this venom and can be produced on a large scale for clinical trials.

## Methods

### Animals

Three adult horses (*Equus caballus*) (400 - 450 kg body mass) maintained in a special animal house at the São Joaquim Farm, Instituto Butantan, São Paulo, Brazil, were used to produce immunoglobulin antivenoms. The horses were vaccinated against the most common equine infectious diseases. 

Adult male Balb/c mice (20-25 g) were obtained from an established colony maintained by the Animal Services Unit of the Universidade Estadual Paulista (UNESP). Mice were housed under controlled humidity at a temperature of 22°C ± 1 and were subjected to a 12-h light-dark cycle in a sound- proofed room. Food and water were available *ad libitum* and mice were taken to the testing room at least 1 h before the experiment. All behavioral testing was performed between 9:00 am and 4:00 pm. All mice were only used once. 

When necessary, animals were anesthetized with a combination of ketamine and xylazine; ketamine is a dissociative anesthetic and xylazine is a powerful sedative/analgesic. In our protocol, a 1:1 mixture of ketamine chloride (Dopalens, Vetbrands, 100 mg/kg of animal) and xylazine chloride (10 mg/kg, Anasedans, Vetbrands) was used. The administration of anesthetics was performed using a 1 mL syringe, with a 23-25 gauge 5/8 inch needle (2 µL/mg bodyweight, intraperitoneally (i.p.)). This was sufficient for surgical procedures lasting from 15 to 30 min or sedation of the animals for 90 mi. After having been used in each experiment, the animals were then euthanized by cervical dislocation under sedation. This procedure was performed only by well-trained personnel.

The experimental protocol was approved by the university’s Committee for Ethics in Animal Experimentation (Science Pharmaceutical School of São Paulo University - protocol 137/07) and followed the ‘‘Principles of Laboratory Animal Care’’ (NIH publication no. 85-23, revised 1985).

### Venom

Africanized honeybees (*Apis mellifera*) were maintained in the Apiary of the Biosciences Institute of UNESP, Rio Claro, SP, southeast Brazil. The honeybee venom was collected using the electric stimulation method (“electric milking”) using a bee venom collector (model VC-6F Apitronics, Richmond, Canada), lyophilized and stored at -20°C until use.

### Antivenom

#### Immunization and bleeding

Africanized honeybee venom antibodies were obtained by immunizing horses by intramuscular (i.m.) injection in the back of each animal of 5 mg of venom in 2.0 mL of incomplete MMT20 adjuvant (Marcol-Montanide-Tween -20 adjuvant, Brazil Ltda-Div. SEPPIC/France/Brazil) mixtures, prepared as described by Herbert (1978) or PBS (phosphate saline, pH 7.5). Fifteen days following the first immunization, the horses received four additional doses of 2.5 mg/2 mL AHB venom at intervals of one week. Two weeks after the last immunization, when the antibodies against the venoms attained an appropriate titer, the horses were bled and a volume of blood corresponding to 2% of their body weight was collected in sterile plastic bags containing anticoagulant. Plasma and cells were separated by gravity sedimentation and the cells were reinfused into the corresponding horse through the jugular vein. Plasma of horses from the same group were pooled and processed. Before immunization, blood samples were withdrawn by jugular vein puncture and the sera were stored at -20°C to be used as negative controls in the antivenom antibody determinations. Three months after bleeding, boosters with similar doses of venom in PBS were given and blood was collected and processed as described. This latter procedure was repeated for six months.

#### F(ab´)_2_ preparation

The protocol for isolating serum rich in F(ab’)_2_ fragments was a minor modification from Pope [13]. A saturated solution of (NH_4_)_2_SO_4_ was added to the plasma pool obtained from the horses immunized with whole AHB venom until a 29% concentration was achieved. The precipitated sediment containing mostly immunoglobulins were separated by continuous centrifugation and dissolved in distilled water. For digestion with pepsin (1 g/80 g protein) the pH was adjusted to 3.0 - 3.2 with 40% (w/v) citric acid and kept under slow stirring at room temperature for 40 min. The pH was then adjusted to 4.5 - 4.6 with 2.5 M NaOH and the lipoproteins were precipitated by the addition of 0.01 M caprilic acid containing 11.3% (w/v) (NH_4_)_2_SO_4_ and incubated at 55°C for 1 h to denature the fibrinogen. A saturated solution of (NH_4_)_2_SO_4_ was added until a final concentration of 17.6% was reached. The mixture was centrifuged and the pellet obtained was discarded. The supernatants were continuously diafiltered on a 30 kDa exclusion membrane (Pellicon System, Merck/Millipore, Darmstadt, Germany) to remove (NH_4_)_2_SO_4_, while the resultant F(ab’)_2_ was concentrated by chromatography on Q-Sepharose Fast Flow anionic exchanger. Briefly, 80 L of F(ab’)_2_ were concentrated through a 20 L chromatography column in phosphate buffer at 25 mM, pH 7.2, at a constant flow of 1850 mL/min, with 1 atm of pressure during 2 h, in a GE industrial system, resulting in a solution containing 98% of F(ab’)_2_. The rich supernatant was concentrated by ultrafiltration through a 10 kDa exclusion membrane and sterilized by passing it through a 0.22 µm filter membrane. 

The preparations were analyzed for the presence of F(ab’)_2_ fragments by comparing their relative molecular weight with uncleaved IgG on sodium dodecylsulfate - polyacrylamide gel electrophoresis (SDS-PAGE). Enzyme-linked immunosorbent assay (ELISA) and Western blot analyses were performed to verify the presence of specific antibodies against ABV antigens. In addition, the solution of F(ab’)_2_ fragments was also submitted to quality control to certify the absence of bacterial contamination and toxic substances. Protein concentrations were determined by biuret assay for equine F(ab’)_2_. The final antivenom concentration was approximately 20 g/L. The protocol for antivenom preparation described above followed the recommendations of the World Health Organization (2010) for this type of product (http://www.who.int/bloodproducts/snake_antivenoms/snakeantivenomguideline.pdf). 

### Immunological assays

#### Elisa

F(ab’)_2_-based antivenom was assayed for its affinity against the proteins and peptides of AHB venom by ELISA. Maxisorp plates (Nalge Nunc, Rochester, NY) were coated with 5 µg venom per well in 0.1 M carbonate buffer, pH 9.6) o/n at 4°C. Unspecific binding was blocked with TBS pH 7.4, 0.05% (v/v) Tween-20 and 1% (w/v) BSA and incubated with F(ab´)_2_ antivenom o/n at 4°C. Bound IgG was detected with alkaline phosphatase-conjugated monoclonal anti-horse IgG antibodies (Sigma Aldrich, Saint Louis, MO). O-phenylenediamine (OPD) was used as substrate (100 µL, 0.1% in 0.07 M citrate buffer, pH 5.0, containing 0.02% H_2_0_2_) and the reaction was stopped after 10 min with H_2_SO_4_ (50 µL, 3 M). The absorbance values at 492 nm were determined using an automated microplate reader (Flow Titertek Multiskan). Unprocessed plasma from non-immunized horse was used as blank. 

## Quality Control Assays

### In Vitro

#### a): Hemolytic assay

The hemolytic assay was based on a method previously described [14]. In summary, 500 µL of mice erythrocytes washed three times with physiological saline solution were suspended in 50 mL of physiological saline solution [0.9% (m/v) NaCl]. Different concentrations of AHB venom (5 - 5000 µg/mL) were incubated with several aliquots of this cell suspension in a final dilution 1:10 at 37°C for 2 h. Samples were then centrifuged and the absorbance of the supernatants measured at 540 nm. The absorbance from lysed red blood cells in presence of 1% (v/v) Triton X-100 was considered 100% and in presence of physiological saline solution as 0%. The hemolytic minimum dose of venom was determined by the minimum quantity of venom that yielded 100% hemolysis. To assess the neutralization efficacy of the antivenom, the dose of neutralizing antivenom was determined as the smallest amount of antivenom able to inhibit 100% of the hemolysis. The results were expressed as the mean ±S.D. of three experiments.

#### b): Cytotoxicity and inhibition of cytotoxicity

 The following cell lines were cultured and tested to determine which was the most sensitive to AHB venom: L-929, a mouse cell line from Adolfo Lutz Institute (|São Paulo, SP- Brazil); CRFK, a feline kidney cell line; MDBK, a Madin-Darby bovine kidney epithelial line from ATCC – CCL-22; and CHO-K1 (Chinese hamster ovary) from ATCC CCL-61.

Venom was serially diluted in 96-well plates using Eagle medium with 10% (v/v) Fetal Bovine Serum (FBS). To determine the best time point, 5 × 10^5^ cells/well were incubated at 36.5°C in 5% (v/v) CO_2_ for 1, 2, 3, 4 or 24 h. Cells were fixed with 80% acetone at -20°C, stained with Blue Evans dye (1:10,000) and incubated for 1 hour at 36.5°C. After staining, cell membranes were disrupted with 10% (w/v) SDS and read at 540 nm to determine the lowest dose of AHB venom able to cause 100% disruption of membrane cells. 

 The cytotoxic dose determined was mixed with different amounts of antivenom and incubated at 36.5°C in presence of 5% CO_2_ for 1 h before performing a cytotoxic test to assess antivenom neutralization capability. Plates were read at 540 nm [15]. 

#### c): Measurement of chondroitin sulfate-degrading activity and its neutralization

 Considering that hyaluronidase also degrades chondroitin sulphate, the activity of this enzyme was assayed based on measurement of the hydrolysis of this substrate using the turbidimetric method described by Long-Rowe [16] with some modifications. Briefly, the reaction medium contained 150 mM calcium chloride, 10 mM sodium acetate buffer pH 5.2 and 0.05 mg/mL chondroitin sulfate in a final volume of 2 mL. After protein addition, the fractions + crude venom were incubated at 37°C for 1 h. The absorbance was measured at 400 nm. One unit of chondroitin sulfate-degrading activity was defined as the amount of enzyme that hydrolyses the release of 2.566 μmol chondroitin sulfate h^-1^ in 1 mL of the reaction medium under the conditions described above. To check the ability of the antivenom to neutralize this activity, the procedure was repeated incubating venom and antivenom for 30 min at 37°C prior to testing. The statistical significance of the results was analyzed using the ANOVA test.

### In Vivo

#### a): Assessment of myotoxic effects by creatine kinase determination

*Preparation procedures* – Mice were prepared as described by Rocha [17]. Briefly, ten Balb/c male mice (5 animals for each group) were anesthetized with a 1:1 mixture of ketamine chloride (Dopalens, Vetbrands, 100 mg/kg of animal) and xylazine chloride (10 mg/kg, Anasedans, Vetbrands) (2 µL/mg bodyweight, i.p.). Fifty micrograms of AHB venom were injected i.m. into the right tibial anterior muscle surgically exposed in a volume of 100 µl of physiological saline solution, and the surgical wound closed. The control group was injected only with saline solution. After 3 h, mice from control and venom groups were again anesthetized, and blood was collected by mandibular vein puncture into heparinized capillary tubes for creatine kinase (CK) measurements. Mice were then euthanized by cervical dislocation under anesthesia. To check inhibition by antivenom, the AHB venom was incubated with antivenom (as determined for neutralization of hemolytic activity) for 30 min at 37°C before performing the test in a group of 5 mice. 

*Serum creatine kinase (CK*)* activity* – Myotoxic activity was quantified by measuring the CK serum activity in both saline- and ABV-injected mice (n = 5 mice/group) using a CK-NAC kit, according to manufacturer’s instructions (Bioclin; Wiener Lab). Immediately after collecting blood by mandibular vein puncture, it was centrifuged (4°C, 3000 rpm/10 min) for serum separation and colorimetric determination of CK activity. The serum CK activity was expressed as the amount of enzyme that will catalyze 1 μmol of substrate per minute under specified conditions. The statistical significance of the results was analyzed using the ANOVA test.

#### b): Hemolytic activity and antivenom neutralization

Assessment of the efficacy of the antivenom for *in vivo* neutralization of the main toxic activity, hemolysis, was performed. Groups of five male Balb/c mice (22-26 g) received an intravenous (i.v.) injection of serial diluted doses of venom starting at 100 µg in 200 µL saline, and blood was collected through mandibular vein after 15 and 30 min, as well as 1, 2, 3, 4, 5 and 6 h to measure the hemolytic activity as described for the *in vitro* determination. The dose resulting in 50% hemolysis *in vivo* (HD_50_) (i.e., the dose that produced a 50% reduction of hematocrit) was determined. 

#### c): Median lethal dose (LD_50_) determinations and neutralization assay

Groups of 5 mice (23–26 g body weight) were injected intravenously, with 0.5 mL of solutions of varying doses of venom dissolved in sterile saline solution. Deaths were recorded after 48 h, and the corresponding LD_50_ was calculated by the Spearman–Karber method (World Health Organization, 1981). As controls, other groups of mice were treated identically, but with 0.2 mL of saline solution instead of venom. 

One venom LD_50_ is defined as the minimal amount of venom causing death in 50% of the mice injected. The test to assess the neutralizing potency of an antivenom is called the median effective dose (ED_50_) assay. 

Determination of the ED_50_ was performed by incubating a fixed amount of venom (“challenge dose”) corresponding to 3-fold LD_50_, with various volumes of the antivenom adjusted to a constant final volume with saline solution (53, 103, 104). The mixtures were incubated for 30 min at 37°C, and then aliquots of 0.5 mL of each mixture were injected into groups of 5 mice (20-25 g) via the i.p. route. A control group injected with a mixture of the venom “challenge dose” with saline solution alone (no antivenom) was included to confirm that the venom “challenge dose” induces 100% lethality.

 The vital signs of all animals used in LD_50_ experiment were monitored during the duration of the experiment; once they became moribund, the animals were euthanized by cervical dislocation under sedation. The animals were monitored every hour in the first 24 h of experiment, and each 2 h from this point until a total of 48 h of observation was achieved. A total of 75 male mice were used, with 5 animals in each group (control, venom treated-group, And Antivenom + Venom Treated Group) Using 5 Dilutions Of Venom Or Antivenom.

## Results and Discussion

There is no specific treatment for victims of multiple bee attacks and there is an urgent need for specific antivenom [6,18]. After a massive bee attack, the victim is submitted to a non-specific treatment, basically consisting of the administration of antihistamines, corticosteroids, bronchodilators, vasodilators, bicarbonate, mannitol, adrenaline and mechanical ventilation, in addition to hemodialysis sessions when needed [8,19].

The antivenom described here was tested against the main toxic activities of AHB venom and it was shown to be efficient in neutralizing hemolysis, cytotoxicity and hyaluronidase activity *in vitro* and also in a number of *in vivo* tests in mice.

**Figure 1:**
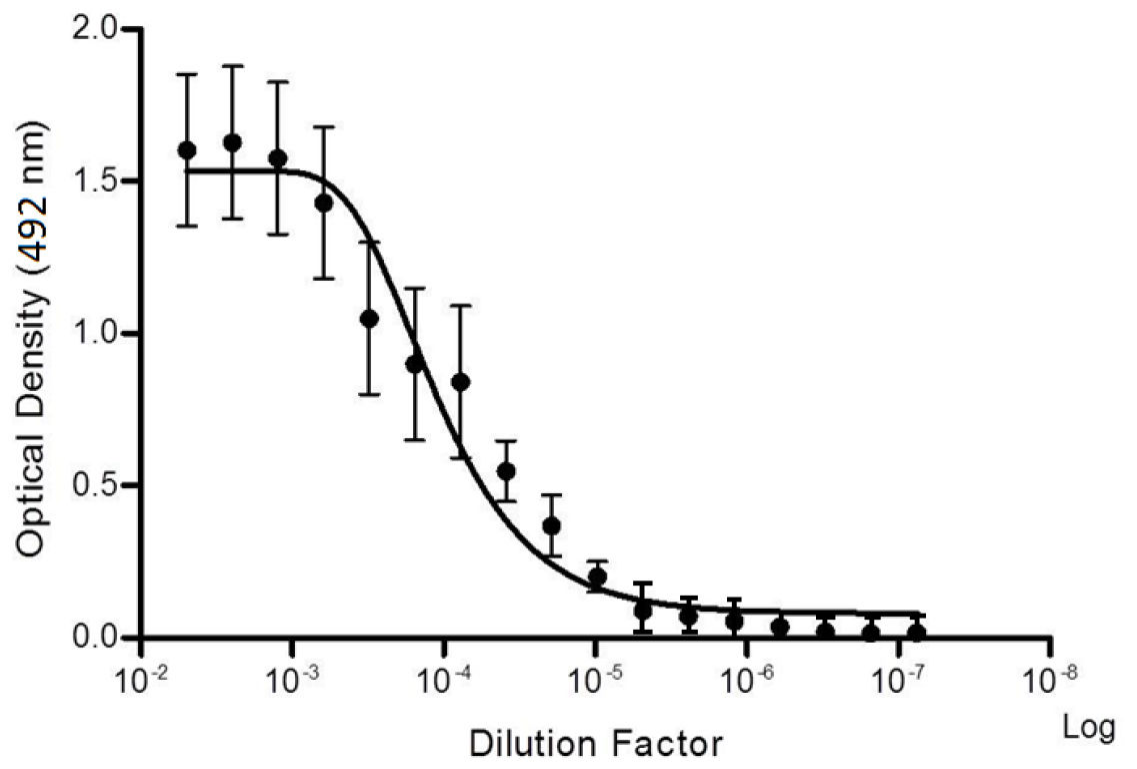
Specific IgG dosage. Specific IgG antisera to Africanized bee venom demonstrated by End Point Titration ELISA.

The immunogenicity of the AHB venom was assessed using the EPT ELISA assay to determine the IgG titer of species-specific IgG antisera (Figure 1). The overall plateau followed by a decline of IgG titer, after successive IgG dilutions, demonstrates that the venom is highly immunogenic (raw data of this assay are shown in Table S1 in File S1).

Some attempts have been made before to develop specific antivenom against bee venom, but even when there was some success, the work did not go further. These attempts were made using Fab fragments [11] or whole IgG [12], and as far as we are concerned, this is the first report of specific bee antivenom using F(ab´)_2_ fragments. The literature concerning the types of fragments used for the production of snake antivenoms is contradictory. Some data on snake antivenoms [20,21] suggest F(ab’)_2_ fragments are better distributed in plasma and neutralize better than F(ab) fragments due to differences in pharmacokinetics. Alternately, other studies have demonstrated that there is no difference between the effectiveness of F(ab’)_2_ and that of whole IgG antivenoms for neutralizing local tissue damage (i.e., the hemorrhage, edema and myonecrosis induced by *Bothrops asper* snake venom in mice) [22]. Considering that the process of production of F(ab')_2_ fragments is a validated method for the production of other antivenoms at Instituto Butantan and since F(ab')2 antivenoms have shown to be effective and to have a good pharmacokinetic profile this was our chosen method for producing AHB antivenom.

**Figure 2:**
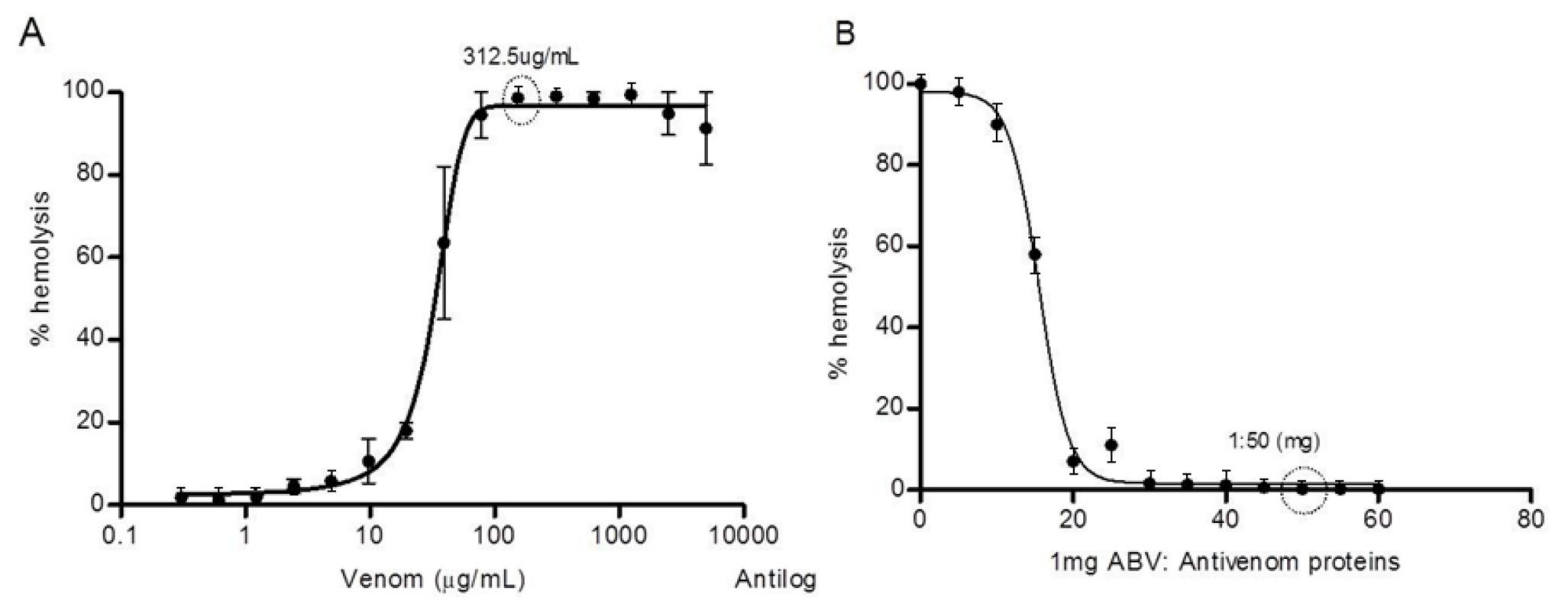
Hemolysis and neutralization of Africanized honeybee (AHB) venom. **A**, Minimum concentration of AHB venom *in* *vitro* able to cause 100% hemolysis was established in 312.5 µg/mL; **B**, Using this established venom concentration the least amount of antivenom needed to completely neutralize the hemolysis was 50-fold times higher than the venom protein content. Values represent average of three experiments.

Hemolysis is the main activity of venom due the high levels of mellitin and phospholipase A_2_, the most abundant components of AHB venom. The minimal amount of AHB venom necessary to cause 100% hemolysis in mice erythrocytes *in vitro* was established at 312.5 µg/mL and used to determine the minimum amount of protein antivenom that would be necessary to neutralize each mg of protein venom (Figure 2) (raw data of these assays are shown in Tables S2 and S3 in File S1). Results showed that the hemolytic action caused by 1 mg AHB venom was neutralized by 2.4 mL antivenom (20.8 mg protein / mL). The antivenom *per se* was not hemolytic, even when used undiluted directly into an erythrocyte suspension (data not shown).

The apparent difficulty thus far has been the raising of antibodies against melittin, the main component of this venom that acts synergistically with PLA_2_ [23]. The main activity of melittin and, therefore whole venom, is hemolysis. As the venom infiltrates blood vessels to enter the circulatory system, phospholipase A_2_ and melittin cause red blood cells to disintegrate. Melittin destroys red blood cells by binding to the cell membrane [23].

**Figure 3:**
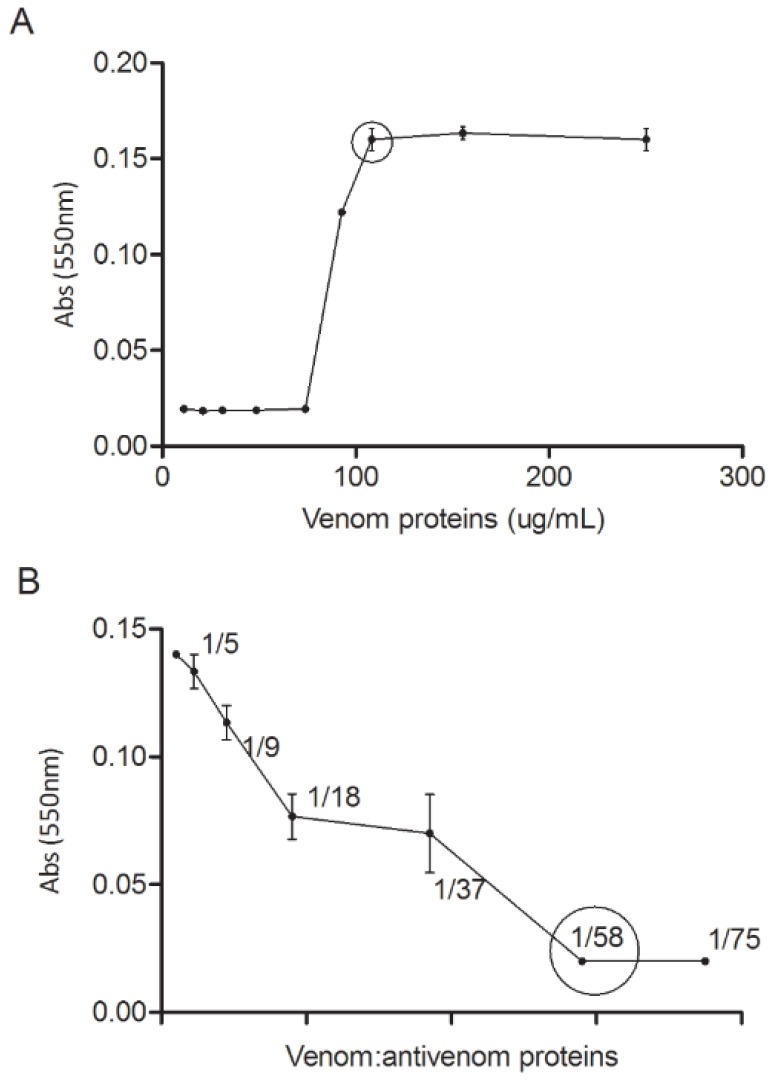
Cytotoxicity of AHB venom and neutralization by antivenom. Cytotoxicity of HBV against CHO cells, highlighting the minimal amount able to cause 100% of cells death - 108.24 mg/mL (**A**); Neutralization of minimal cytotoxic dose by diluted antivenom - 1:58 (**B**). Values represent average of three experiments.

Whole bee venom has been demonstrated to have cytotoxic effects on HL-60 cells and human lymphocytes [24]. Among the different cell lines tested, CHO cells were found to be the most sensitive to AHB venom, thus smaller amounts of venom were necessary to cause cytotoxicity. Antivenom was able to fully neutralize the cytotoxic activity of venom in CHO cells (Figure 3), meaning that the cytotoxic action of 1 mg of whole AHB venom on CHO cells were neutralized by 0.57 mL of antivenom (raw data of this assay are shown in Table S4 in File S1). 

The incubation of venom/antivenom mixtures remains the most frequently used method for the determination of the antivenom neutralizing potential. Usually, the curve is not linear, as is observed for the neutralization of cytotoxic and hemolytic effects. This suggests that a toxic component, regardless of the amount present in the venom, may be a poor antigen and induces only low levels of neutralizing antibodies [25,26]. This could be the case of melittin in AHB venom and could explain the failure to neutralize hemolysis in previous attempts.

**Figure 4:**
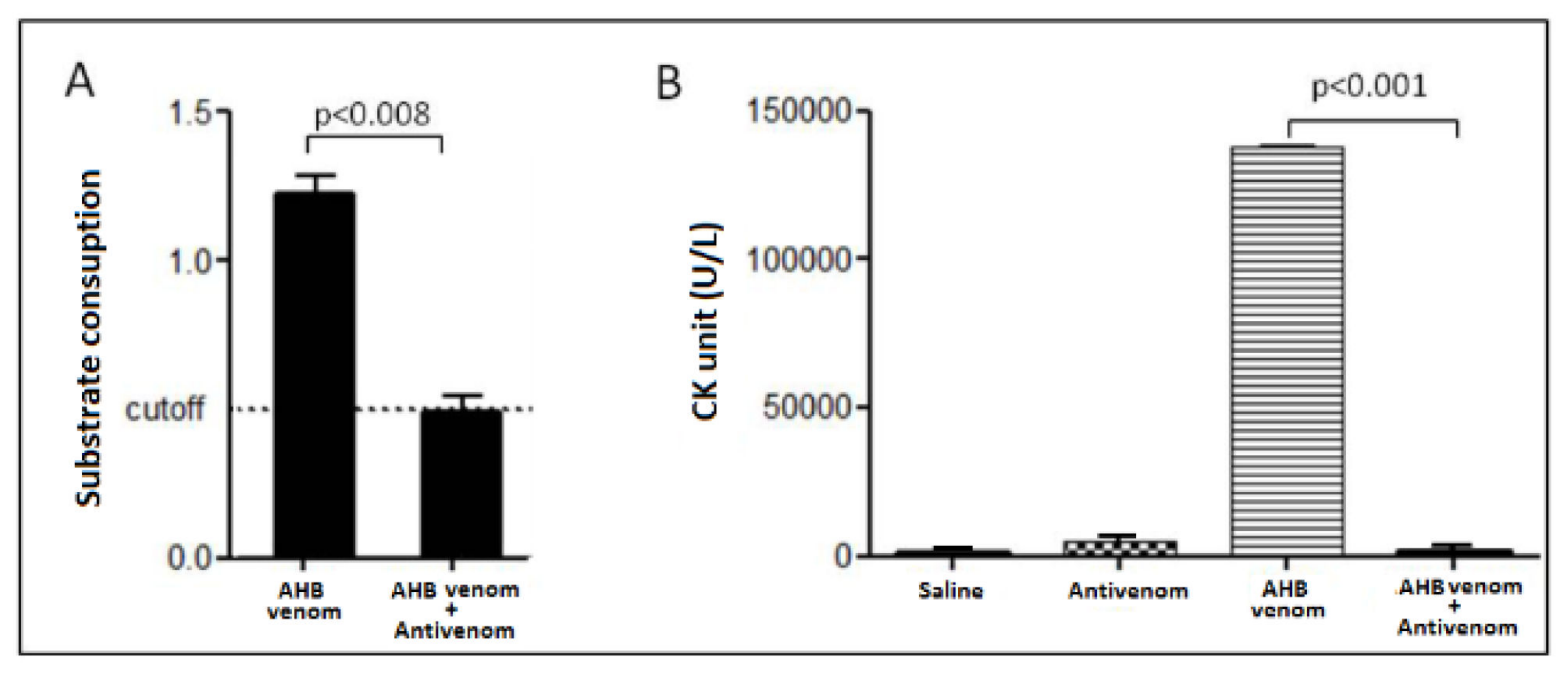
Inhibition of hyaluronidase *in* *vitro* and myotoxic activity of AHB venom *in* *vivo*. **A**, The activity of hyaluronidase is shown for the whole venom as well for the dialyzed venom using the same protein content. The inhibition of this enzyme by antivenom was completely achieved. Values represent an average of three experiments. **B**, The myotoxic activity was measured by creatine kinase release and expressed the amount of enzyme that will catalyze 1 μmol of substrate per minute under specified conditions. The specific antivenom was able to completely neutralize this activity *in* *vivo*. Values represent the average of three experiments. Statistical analysis – ANOVA. CK – Creatine kinase; AV – antivenom; AHBV – Africanized bee venom.

 Hyaluronidase, the third most important component in bee venom (after melittin and phospholipase A_2_) [27] was also fully inhibited by antivenom that was not harmful even when administered undiluted (Figure 4A). Honeybee antivenoms previously reported [11,12] were not tested against hyaluronidase, the second most abundant enzyme in the venom. Although hyaluronidase is not toxic enough to cause death, this enzyme has an important role in the first steps of envenomation process. Hyaluronidase breaks down hyaluronic acid present in the extracellular matrix. This enables the venom to spread into tissues more easily [4,11]. 

 To verify the myotoxicity neutralization ability of antivenom *in vivo*, we measured the release of creatine kinase in mouse muscle. This effect can be evaluated by morphological analysis and by monitoring the increase of plasma creatine kinase (CK) activity. The increase in plasma CK levels results from sarcolemmal damage due to myotoxic components of the venom, such as PLA_2_ and melittin [28]. The results displayed in Figure 4B confirm that specific antivenom was able to completely neutralize the myotoxic effects and was not harmful when injected undiluted in mice (raw data of these assays are shown in Tables S5 and S6 in File S1).

**Figure 5:**
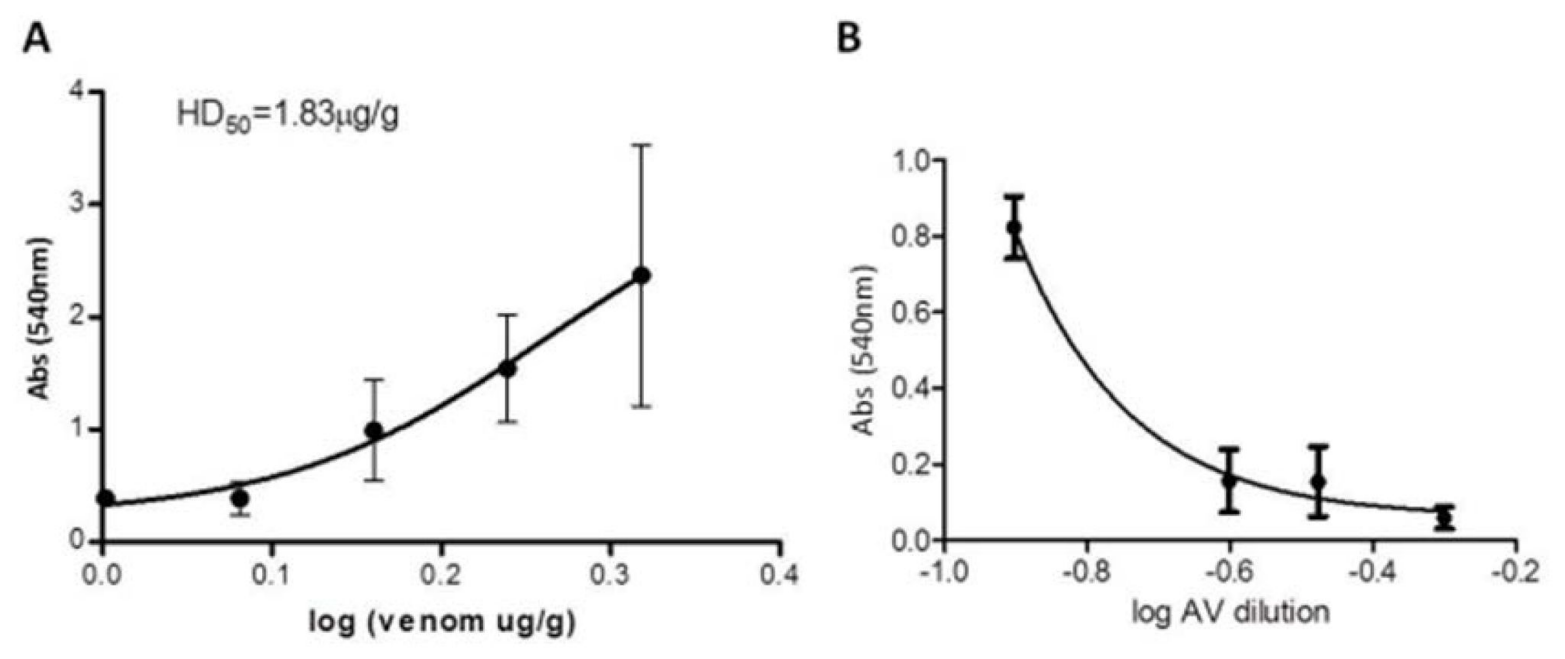
Hemolysis and neutralization *in* *vivo*. **A**, Determination of Hemolytic Dose 50% (HD_50_) collecting blood after 15 min of AHB venom injection in mice. **B**, Neutralization of hemolytic activity using 3-fold times HD_50_ (5.49 µg/g) and different dilutions of antivenom collecting blood after 15 min of AHB venom injection in mice. Higher values of absorbance at 540 nm indicate higher hemolytic activity.

 Because hemolysis is the most prominent toxic activity of honeybee venom, we determined the median hemolytic dose (HD_50_) and thus the antivenom-neutralizing effective dose (ED_50_) *in vivo*. The peak time for hemolysis was 15 min. The HD_50_ was determined in 1.83 µg venom / g of mouse body weight (Figure 5A). An amount equivalent to 3-fold times the HD_50_ value (5.49 µg/g) was used to determine neutralization of hemolytic activity *in vivo*. The neutralization of hemolysis was expressed as an effective dose (ED_50_), defined as 0.03304 mg/mL (Figure 5B) that is the amount of AHB venom (expressed in mg) which was neutralized by an amount of antivenom (expressed in mL) (Figure 5B) (raw data of these assays are shown in Tables S7 and S8 in File S1). The potency of antivenom was calculated considering: 

Potency of antivenom = total venom used in test - 1EC50/EC_50_

Potency of antivenom = 5.49 - 1.83/0.03304 = 110.77 ug/mL or 0.11mg/mL.

As an essential prerequisite and ultimate test to assess antivenom efficacy, we determined the effect of the neutralization antivenom on the LD_50_. The lethal potency of bee venom i.v. injected was 4.2 ± 0.2 µg / g of body weight. Experimentally, the lethal action caused by 1 mg AHB venom was neutralized by 0.9 mL antivenom meaning ED_50_ is 1.11mg/mL antivenom . Making a rough extrapolation based on these results, in a massive bee attack involving 1000 stings (dose considered lethal for a 70 kg adult man) [29] and that each bee is able to inject approximately 100 µg of venom [30], it would require 90 mL of this antivenom to neutralize the venom’s lethal effects. However the dose to be used in clinical human cases should be determined in a dose-finding clinical trial.

At this time it is important to emphasize that despite to have administered venom through intravenous route for determining LD_50_, the neutralization by antivenom was performed by intraperitoneal application. This occurred because the volume of antivenom was relatively high in relation to the blood volume of the animal, and could cause embolism, which could result in false toxicity. Anyway, despite the value of LD_50_ by i.p. route was not shown, the group of animals which was treated only with venom died, while the group that also received the antivenom survived the envenomation.

High levels of bee venom could be detected in the circulation of multiple-sting victims that were receiving basic support treatment but died even 53 h after a massive bee attack [8], which could most likely be neutralized by the use of a specific antivenom. Although in such accidents, specific antivenom should be administered as soon as possible, there is evidence suggesting that specific antivenom should be very useful even hours after the stinging incident [11,32]. The consequences of a systemic reaction following a massive bee attack can only be noticed several hours after accident, with deaths occurring from 22 to 72 h after an attack [8,10].

A report demonstrating the procedures adopted by some hospitals in the US recommends a 6-hour observation period for all patients who sustain 50 or more stings but are experiencing no symptoms or experiencing just pain [10]. Additionally, it is suggested that this period should be extended to 24 h for elderly or pediatric because these patients have an increased risk of tissue injury that may be delayed and may be more effectively treated if identified early rather than as on 12-to 24-hour follow-up [31].

European bee venom has been shown to be very similar in composition to AHB venom [11], indicating that it is probable that this antivenom can effective neutralize also venom from European sub-species of *A. mellifera.*

The antivenom production and characterization reported here was produced at Butantan Institute, the world's largest producer of snake antivenom following strict international guidelines for production of antivenoms. This AHB antivenom will hopefully be an efficient product and its large-scale production could be used in clinical trials. 

A direct comparison between antiofidic antivenom and this bee antivenom should not be made in terms of mean potency, because the immunogenicity of these venoms is different. Honeybee venom is not primarily lethal, but in high amounts causes morbidity and eventually can lead to death. Furthermore the amount of venom injected even by a massive attack of bees is far smaller than the amount of venom injected by a venomous snake what justify the production of the bee antivenom even if it has a lower potency when compared to some snake antivenoms. 

After the positive results presented here, the produced antivenom will be submitted to clinical trials under the supervision of physicians at the Hospital Vital Brazil from Instituto Butantan, São Paulo, Brazil, to certify its neutralization capabilities and to establish the correct dose regimen. Based on these results, the Butantan Institute will scale-up the production and raise a final product that can be distributed to areas where there is a greater risk of accidents.

## Conclusions

We report here the first generation of a hyperimmune F(ab’)_2_-based equine specific AHB antivenom that is efficient to neutralize the main toxic effects of this venom *in vitro* and also *in vivo* in mice presenting an effective dose of 0.9 mg/mL. The type of IgG fragment produced and the procedure for monitoring neutralization of hemolytic activity were used here for the first time to assess the efficacy of the AHB venom, which mainly distinguishes it from the previous antivenoms reported in the literature [11,12]. 

The final product was approved in preclinical assays in quality, safety and efficacy, the most important criteria of antivenom production. 

The results herein provide promising evidence that this specific antivenom can be the first efficient treatment against massive honeybee attacks.

## Supporting Information

File S1 **Supporting Information**.
Table S1, Raw data for Figure 1: Specific IgG antisera to Africanized bee venom demonstrated by End Point Titration ELISA. Table S2, Raw data for Figure 2A: Hemolysis and neutralization of Africanized bee venom (ABV). Table S3, Raw data for Figure 2B: Hemolysis and neutralization of Africanized bee venom (ABV). Table S4, Raw data for Figure 3: Cytotoxicity of ABV and neutralization by antivenom. A) Venom proteins; B) Venom:antivenom proteins. Table S5, Raw data for Figure 4A: Inhibition of hyaluronidase in vitro and myotoxic activity of ABV in vivo. Table S6, Raw data for Figure 4B: Inhibition of hyaluronidase in vitro and myotoxic activity of ABV in vivo. Table S7, Raw data for Figure 5A: Hemolysis and neutralization in vivo. Table S8, Raw data for Figure 5B: Hemolysis and neutralization in vivo.
(DOC)
