# Supplementary material for: Production of the First Effective Hyperimmune Equine Serum Antivenom against Africanized Bees
Source: PLoS One. 2013 Nov 13;8(11):e79971. doi: 10.1371/journal.pone.0079971 (PMC3827448; doi:10.1371/journal.pone.0079971)
Supplement: File S1 — Supporting Information. Table S1, Raw data for Figure 1: Specific IgG antisera to Africanized bee venom demonstrated by End Point Titration ELISA. Table S2, Raw data for Figure 2A: Hemolysis and neutralization of Africanized bee venom (ABV). Table S3, Raw data for Figure 2B: Hemolysis and neutralization of Africanized bee venom (ABV). Table S4, Raw data for Figure 3: Cytotoxicity of ABV and neutralization by antivenom. A) Venom proteins; B) Venom:antivenom proteins. Table S5, Raw data for Figure 4A: Inhibition of hyaluronidase in vitro and myotoxic activity of ABV in vivo. Table S6, Raw data for Figure 4B: Inhibition of hyaluronidase in vitro and myotoxic activity of ABV in vivo. Table S7, Raw data for Figure 5A: Hemolysis and neutralization in vivo. Table S8, Raw data for Figure 5B: Hemolysis and neutralization in vivo. (DOC) [file pone.0079971.s001.doc]

**PRODUCTION OF THE FIRST EFFECTIVE HYPERIMMUNE EQUINE SERUM ANTIVENOM AGAINST AFRICANIZED BEES**

Keity Souza Santos^a,b,i^, Marco Antonio Stephano^c^, José Roberto Marcelino^d^, Virginia Maria Resende Ferreira^a,b^, Thalita Rocha^e^, Celso Caricati^f^, Hisako Gondo Higashi^g^, Ana Maria Moro^b,h^, Jorge Elias Kalil^a,b,i^, Osmar Malaspina^b, j^, Fabio Fernandes Morato Castro^a,b,i^, Mário Sérgio Palma^b, j^ *

^a^ Division of Clinical Immunology and Allergy, University of São Paulo School of Medicine, FMUSP, São Paulo, Brazil

^b^ Institute for Investigation in Immunology–INCT, São Paulo - SP, Brazil

^c^ Faculdade de Ciências Farmacêuticas, São Paulo University, São Paulo - SP, Brazil

^d^ Division of Hyperimmune Plasmas, Butantan Institute, São Paulo - SP, Brazil

^e^ Medical School - São Francisco University - Bragança Paulista - SP, Brazil

^f^ Special Pilot Laboratory of Research and Development of Veterinary Immunobiologicals,

Butantan Institute, São Paulo - SP, Brazil

^g^ Institute of Technology of Paraná, Curitiba, PR, Brazil

^h^ Lab of Biopharmaceuticals in Animal Cells, Butantan Institute, São Paulo - SP, Brazil

^i^ Heart Institute (InCor), LIM-19, University of São Paulo School of Medicine, São Paulo-SP,

Brazil

^j^ Institute of Biosciences/Department of Biology, Center for the Study of Social Insects,

University of São Paulo State (UNESP), Rio Claro - SP, Brazil

**Table S 1 – Raw data for Figure 1: Specific IgG antisera to Africanized bee venom demonstrated by End Point Titration ELISA.**

Dilution Factor Optical Density (405 nm)

0.005000 1.852000 1.352000 1.650000

0.002500 1.877667 1.377667 1.677870

0.001250 1.826333 1.326333 1.520000

0.000625 1.679000 1.179000 1.579000

0.0003125 1.298500 0.798500 1.009850

0.00015625 1.149667 0.6496667 0.956000

0.000078125 1.090500 0.590500 0.850000

0.0000390625 0.6476667 0.4476667 0.508700

0.0000195313 0.4676667 0.2676667 0.409800

0.00000976563 0.251000 0.151000 0.300000

0.00000488281 0.08966666 0.08966666 0.151000

0.00000244141 0.071000 0.071000 0.089667

0.0000012207 0.054500 0.054500 0.071000

0.000000610352 0.035000 0.035000 0.055500

3.051760e-007 0.020000 0.020000 0.018000

1.525880e-007 0.01366667 0.01366667 0.010000

7.629390e-008 0.01633333 0.01633333 0.013667

**Table S2 – Raw data for Figure 2A: Hemolysis and neutralization of Africanized bee venom (ABV).**

**Venom (ug/mL) % hemolysis % hemolysis % hemolysis**

5000.000000 82.631580 100.000000 100.000000

2500.000000 89.736840 100.000000 100.000000

1250.000000 98.947360 100.000000 89.000000

625.000000 96.842100 100.000000 95.000000

312.500000 100.000000 98.000000 97.000000

156.250000 100.000000 97.000000 100.000000

78.125000 100.000000 89.000000 100.000000

39.063000 45.000000 82.000000 100.000000

19.531000 16.052630 20.000000 12.052630

9.765500 5.263158 16.052630 5.263158

4.882750 6.315790 5.263158 5.263158

2.441375 2.894737 6.315790 5.263158

1.220688 1.052632 2.894737 2.105263

0.6103438 1.578947 1.052632 0.578947

0.3051719 2.105263 1.578947 0.578947

**Table S 3 – Raw data for Figure 2B: Hemolysis and neutralization of Africanized bee venom (ABV).**

**1mg ABV: Antivenom proteins % hemolysis % hemolysis % hemolysis**

0 100.0 98.947360 100.000000

5 98.0 96.842100 100.000000

10 90.0 100.000000 98.000000

15 88.0 100.000000 97.000000

20 70.0 100.000000 89.000000

25 51.0 45.000000 82.000000

30 1.6 16.052630 20.000000

35 1.3 5.,263158 16.052630

40 1.1 6.315790 5.263158

45 0.6 2.894737 6.315790

50 0.2 1.052632 2.894737

55 0.2 1.578947 1.052632

60 0.2 2.105263 1.578947

**Table S4 – Raw data for Figure 3: Cytotoxicity of ABV and neutralization by antivenom.**

**A)**

**Venom proteins (ug/mL) Abs at 550nm Abs at 550nm Abs at 550nm**

11.02 0.020 0.018 0.020

20.95 0.018 0.019 0.018

31.08 0.019 0.018 0.019

48.52 0.018 0.020 0.018

73.88 0.020 0.018 0.020

92.69 0.120 0.121 0.125

108.24 0.160 0.150 0.170

155.49 0. 160 0.170 0.160

250,.32 0.160 0.150 0.170

**B)**

**Venom:antivenom proteins Abs at 550nm Abs at 550nm Abs at 550nm**

2.0 0.14 0.14 0.14

4.5 0.14 0.14 0.12

9.0 0.10 0.12 0.12

18.0 0.08 0.06 0.09

37.0 0.04 0.08 0.09

58.0 0.02 0.02 0.02

75.0 0.02 0.02 0.02

**Table S5 – Raw data for Figure 4A: – Inhibition of hyaluronidase in vitro and myotoxic activity of ABV in vivo.**

**AHB Venom:** 1.300 1.100 1.272

**AHB Venom + Antivenom:** 0.400 0.500 0.582

**Table S6 – Raw data for Figure 4B: Inhibition of hyaluronidase in vitro and myotoxic activity of ABV in vivo.**

**Saline:** 1396.38 1500.00 1394.30

**Antivenom** : 5322.46 5320.20 5324.00

**AHB Venom:** 138181.70 138182.90 137000.00

**AHB Venom + Antivenom:** 1821.37 1750.00 1820.00

**Table S7– Raw data for Figure 5A: Hemolysis and neutralization in vivo.**

**log (venom ug/g) Abs (540nm) Abs (540nm) Abs (540nm)**

2.08 0.3180 1.996 2.222 2.895

1.736 0.2390 1.720 1.566 1.338

1.446 0.1600 1.172 0.991 0.813

1.205 0.0810 0.334 0.453 0.382

1.004 0.0017 0.417 0.383 0.370

**Table S8– Raw data for Figure 5B: Hemolysis and neutralization in vivo.**

**Dilution log AV dilution Abs (540nm) Abs (540nm) Abs (540nm)**

1/2 -0.30103 0.017 0.003 0.103

1/3 -0,47756 0.334 0.031 0.100

1/4 -0.60206 0.400 0.120 0.061

1/8 -0.90309 0.683 0.685 0.925
